# Myocardial Late Gadolinium Enhancement (LGE) in Cardiac Magnetic Resonance Imaging (CMR)—An Important Risk Marker for Cardiac Disease

**DOI:** 10.3390/jcdd11020040

**Published:** 2024-01-26

**Authors:** Claudia Meier, Michel Eisenblätter, Stephan Gielen

**Affiliations:** 1 Universitätsklinik für Kardiologie, Angiologie und Internistische Intensivmedizin, Universitätsklinikum Ostwestfalen-Lippe, Campus Klinikum Lippe, D-32756 Detmold, Germany; 2 Medizinische Fakultät, Universität Bielefeld, D-33615 Bielefeld, Germany; 3 Universitätsinstitut für Diagnostische und Interventionelle Radiologie, Universitätsklinikum Ostwestfalen-Lippe, Campus Klinikum Lippe, D-32756 Detmold, Germany

**Keywords:** late gadolinium enhancement, cardiac magnetic resonance imaging, cardiomyopathy, myocardial vitality, risk stratification, review

## Abstract

Cardiovascular magnetic resonance (CMR) has significantly revolutionized the comprehension and diagnosis of cardiac diseases, particularly through the utilization of late gadolinium enhancement (LGE) imaging for tissue characterization. LGE enables the visualization of expanded extracellular spaces in conditions such as fibrosis, fibrofatty tissue, or edema. The growing recognition of LGE’s prognostic capacity underscores its importance, evident in the increasing explicit recommendations within guidelines. Notably, the contemporary characterization of cardiomyopathies relies on LGE-based scar assessment by CMR to a large extent. This review describes the pattern and prognostic value of LGE in detail for various cardiac diseases. Despite its merits, establishing LGE as a reliable risk marker encounters challenges. Limitations arise from the fact that not all diseases show LGE, and it should always be analyzed in the context of all CMR sequences and the patient’s medical history. In summary, LGE stands as a robust indicator of adverse outcomes in diverse cardiovascular diseases. Its further integration into routine practice is desirable, necessitating widespread availability and application to accumulate both individual and scientific experience.

## 1. Introduction

Cardiovascular magnetic resonance (CMR) has transformed the understanding and diagnostic pathway of various cardiac diseases, particularly with the use of late gadolinium enhancement (LGE) imaging for tissue characterization. In addition, the prognostic ability of LGE to predict outcome is becoming increasingly important, and that has been reflected by the growing numbers of explicit recommendations in the guidelines of the last years [1,2,3]. Furthermore, the new ESC guidelines for the management of cardiomyopathies [1] establish their new phenotypic description of cardiomyopathies on a LGE-based scar assessment by CMR. Especially in cardiomyopathies, traditional risk factors do not adequately predict outcome as there is no pathogenetic correlation. The presence, location, pattern, and extent of LGE has been shown to be a prospective and innovative risk marker for the development of a symptomatic phenotype and adverse events. The aim of this review is to highlight the role of LGE as a risk marker for various cardiovascular diseases.

When considering LGE as a “risk factor” for the development or exacerbation of heart disease, it should be noted that the presence of LGE itself does not cause the disease, but is a surrogate for certain physiological or anatomical characteristics, genetic predisposition or metabolic constellations that represent a risk. So, the term “risk marker” should be preferred. The use of the chelated paramagnetic contrast agent gadolinium within a CMR scan can visualize the widened extracellular space in, e.g., fibrosis, fibro-fatty tissue, or edema. This technique exploits differences in gadolinium washout kinetics and volume of distribution between scar or edematous tissue and normal myocardium. Gadolinium shows rapid washout from healthy myocardium. In contrast, it washes out more slowly from areas of fibrosis or edema where the extracellular space is enlarged. T1-weighted inversion recovery sequences, optimized to “null” the signal from healthy myocardium, reveal areas of, e.g., scar or edematous tissue as bright regions of high signal intensity by shortening T1 relaxation times.

## 2. LGE in Specific Phenotypes of Cardiovascular Disease

Different heart diseases cause a different pattern and a different extent of LGE, which is why the distribution pattern and the total burden of LGE as a risk marker must be considered separately for each disease and cannot be generalized. Furthermore, it must be noted that any change in interstitial space from any cause can be a potential origin of LGE. So, the common perception that LGE is synonymous with fibrosis and therefore dead tissue is too simplified.

It must be also emphasized that the interpretation of CMR images is only conclusive when all morpho-functional images (cine-imaging, perfusion-imaging) and all tissue-characterizing images (T1 weighted, T2 weighted, possibly multiparametric mapping, possibly T2 * weighted) are taken together and that the LGE cannot be interpreted on its own. Please note that the significance of the LGE was highlighted for this overview.

### 2.1. Ischemic Cardiomyopathy

Ischaemic cardiomyopathy (ICM) is a sub-entity of heart failure with reduced ejection fraction in which a mismatch between myocardial oxygen demand and vascular oxygen supply leads to reversible or irreversible myocardial damage. The majority of patients with ICM has experienced either a type I myocardial infarction following a plaque rupture and thrombus in epicardial conduction vessels or a type II myocardial infraction resulting from vasospasm, microvascular dysfunction, non-arteriosclerotic coronary dissection or regional non-obstructive relative ischemia. This is still the predominant cause of heart failure globally [4,5,6]. It typically results from a combination of irreversible loss of viable myocardial mass and a dysfunctional but still viable myocardium in the setting of chronically reduced myocardial blood flow.

**Figure 1:**
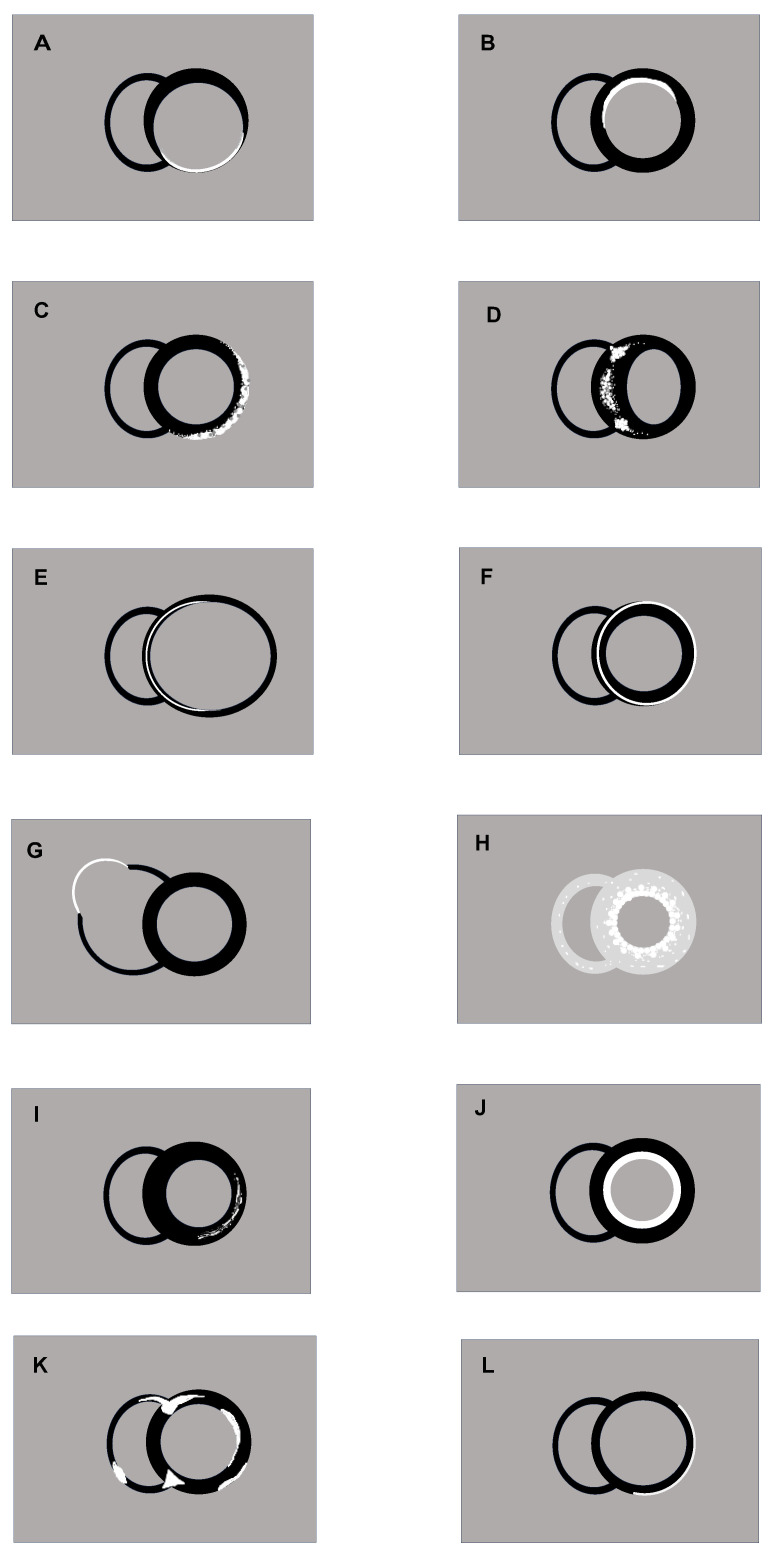
Scheme of typical LGE patterns in short axis view, T1-weighted inversion recovery sequence; black: myocardium without LGE, white: LGE. Please note: the distribution pattern may vary within certain limits and should only be assessed in the context of patient’s history and the whole CMR examination. (**A**): Transmural ischemic scar with myocardial thinning as consequence of RCA-Infarction. (**B**): Non-transmural ischemic scar as consequence of LAD-infarction. (**C**): Myocarditis with subepicardial inferolateral LGE. (**D**): Classical hypertrophic cardiomyopathy with LGE at the RV insertions and in the area of greatest hypertrophy. (**E**): Dilated cardiomyopathy with a fine line of intramural septal LGE. (**F**): Non-dilated cardiomyopathy with ring-like LGE and a septal intramural and lateral epicardial distribution. (**G**): Classical ARVC with right ventricular dilatation and aneurysms with LGE. (**H**): Cardiac amyloidosis with strong LGE originating from the subendocardium in the hypertrophied LV and RV myocardium. (**I**): Anderson–Fabry disease with mild intramural to subepicardial inferolateral LGE and hypertrophy. Note: relatively frequent, unspecific pattern, also possible with increasing pressure load or as post-inflammatory residual. Additional T1 mapping sequences required for differentiation. (**J**): Endomyocardial fibrosis with LGE in the thickened endocardium, here without thrombosis. (**K**): Cardiac sarcoidosis with patchy subendocardial, subepicardial or transmural distribution, anterior “Hook-sign” and inferior “Triangle sign” in place of the RV insertions. (**L**): Cardiac involvement in muscular dystrophy Duchenne with subepicardial lateral LGE in thinned myocardium. Note: also possible in DCM of other origin.

**Figure 2:**
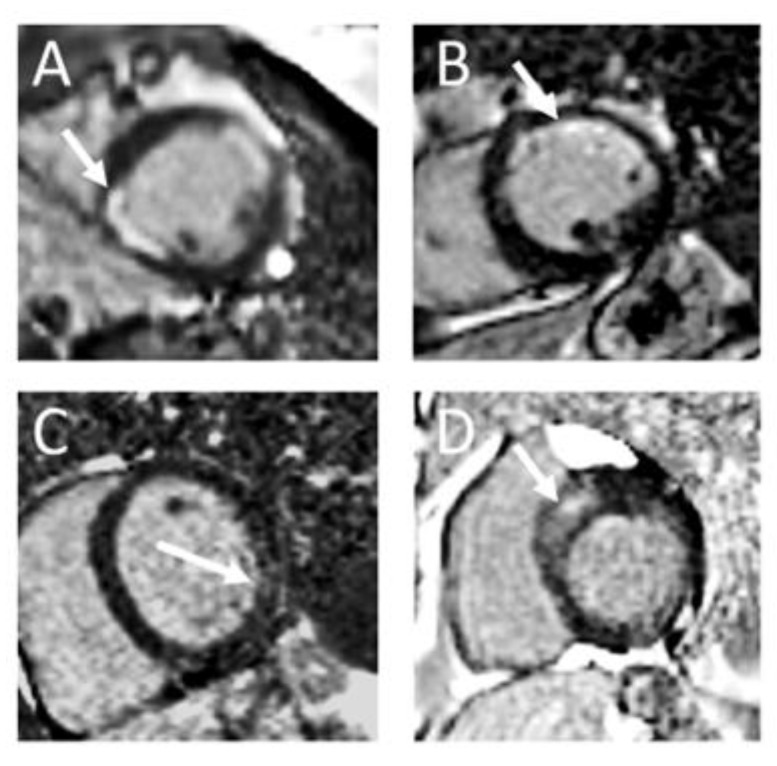
Illustrative examples of LGE in important cardiac diseases: (**A**): Patient with s/p ST-Elevation myocardial infarctions in anterior and inferolateral location with LGE >50% of myocardial wall thickness. (**B**): Patient with s/p non-ST-Elevation myocardial infarction in anterior location with subendocardial LGE < 50% of myocardial wall thickness. (**C**): Patient with viral myocarditis and patchy lateral LGE. (**D**): Patient with hypertrophic cardiomyopathy (HCM) and septal LGE in the area of greatest hypertrophy.

The typical distribution of subendocardial LGE corresponding to a coronary artery territory identifies an ischemic scar, whereby the transmurality indicates the residual vitality [7] (Figure 1A,B and Figure 2A,B). A transmural infarction affects all wall layers from the endocardium to the epicardium, whereas a non-transmural infarction originates from the endocardium and affects <100% of the wall thickness. The size of the infarction, evaluated through LGE-CMR, stands out as the most robust predictor of mortality and significant cardiac events. Not only the infarct transmurality, but also total scar mass, total scar as a percentage of LV volume, gray zone mass, and the peri-infarction-to-core infarction mass ratio are important for risk stratification [8]. So, CMR is also effective in assessing myocardial vitality through discrimination of the LGE extension and segmental kinesis and this can guide coronary revascularization [9]. There is a close relation between the percentage of the left ventricular wall thickness which is affected by the infarction scar and functional recovery after myocardial revascularization: wall segments with <25% LGE extension are more likely to regain contractility than segments with >50% LGE transmurality [10].

Guidelines recommend that CMR with LGE should be performed as soon as possible in patients with suspected MINOCA (Myocardial Infarction with Nonobstructive Coronary Arteries). The ischemic pattern in the absence of invasive stenosis reliably ensures the diagnosis [6,11,12]. In addition to its diagnostic value, the extent of LGE has been shown to have prognostic significance in MINOCA [13]. According to data from the SPINS registry, a greater extent of ischemic burden in ischaemic cardiomyopathy in general was associated with an increased risk of major cardiac events, including hospitalization for congestive heart failure [14,15]. In addition, the presence of transmural necrosis has been shown to correlate with responses to cardiac resynchronization therapy and the risk of arrhythmias [16,17,18]. In summary, LGE in ischemic cardiomyopathy is able to confirm the diagnosis, guide therapy, and predict outcome.

Central foci of low signal or hypoenhancement within the LGE of a myocardial infarction represent areas of microvascular obstruction (MVO) affecting vessels smaller than 200 μm, and are known angiographically as no-reflow zones. Typically, gadolinium penetrates slowly into the damaged capillaries, as evidenced by increasing whitening on very late sequences. This phenomenon is associated with a poor prognosis and serves as a marker for subsequent adverse left ventricular remodeling [19].

### 2.2. Myocarditis

Patients suspected of having myocarditis exhibit a diverse range of clinical presentations, making the diagnosis, monitoring, and prognosis challenging. While viruses are the primary cause of myocarditis, it can also be induced by factors such as drugs (e.g., checkpoint inhibitors [20]), toxic substances, or autoimmune conditions [21]. CMR diagnostics became particularly important in the time of COVID-19 associated myocarditis [22,23,24].

The first in 2009 published Lake Louise criteria (LLC) [25] for diagnosing acute myocarditis used specific tissue characteristics in CMR, including LGE imaging. The LCC have been updated recently [26] and now also include novel imaging techniques such as T1 and T2 mapping and ECV calculation. CMR has largely replaced endomyocardial biopsy, the gold standard of diagnosis, and extensive LGE has proven to increase the risk of adverse outcome [27]. In this context, LGE cannot be interpreted alone and especially T2 STIR sequences and T2 mapping are sensitive in detecting acute states of myocarditis by edematous water deposition in the extracellular space. It could even be shown that CMR was able to change the initial suspected diagnosis of acute myocarditis (which was created according to the 2013 European Society of Cardiology position statement criteria for clinically suspected myocarditis [28]) to another diagnosis in almost 20% of cases [29].

Myocarditis often shows a subepicardial patchy pattern of LGE, predominantly in the basal inferolateral wall, although other locations do not exclude this diagnosis (Figure 1C and Figure 2C). In the acute phase of inflammation, the extent of the LGE is greater than in the healed residual and may even disappear completely. This is because acute oedema is also a cause of extracellular space expansion and not just irreversible cell damage. In the case of pronounced myocarditis, a CMR follow-up is recommended after approx. 3–6 months to assess whether the acute reaction has subsided and to evaluate the final extent of the scar burden [30]. The extent of LGE has been shown to be negatively correlated with left ventricular function and could predict improvement in follow-up [31]. In addition, LGE has been shown to be a tool for predicting outcome in myocarditis [31,32].

### 2.3. Hypertrophic Cardiomyopathy

CMR significantly helps to distinguish the different subtypes of the heterogeneous group of diseases with a hypertrophic phenotype, which previously could only be clarified by biopsy. The classic phenotype of hypertrophic cardiomyopathy (HCM) is an asymmetric, septal hypertrophy with (HOCM) or without obstruction of the outflow tract, caused by an autosomal dominant mutation in the sarcomere genes with a prevalence of about 1:350 [33]. CMR is especially important in the detection of midventricular or apical variants of HCM, in which echocardiographic evaluation has limitations [34]. In addition, CMR can detect myocardial crypts [35] and papillary muscle abnormalities [36], which may be a subclinical marker of HCM, and depict apical aneurysms with and without thrombus formation, which is considered a major risk factor in the American College of Cardiology/American Heart Association (ACC/AHA) [37] and ESC [2] guidelines, leading to recommendations for implantable cardioverter-defibrillator (ICD) implantation.

LGE in HCM signifies replacement fibrosis, and its prognostic significance is well-established. It is detected in over 50% of HCM patients, typically manifesting as a mid-mural pattern within the most hypertrophied segments [38] and on right ventricular insertion points (Figure 1D and Figure 2D). In the advanced stages of the disease, LGE with transmural extension may be observed, carrying a poorer prognosis, because the extent of LGE consistently correlates with an increased incidence of sudden cardiac death [39]. In a pivotal multicenter study, the presence of LGE exceeding 15% of the left ventricular (LV) mass was associated with a more than two-fold risk of SCD in patients initially classified as low risk by conventional tools, compared to patients without LGE [38]. Consequently, the presence of “extensive LGE” (≥15% of total LV mass) is considered a high-risk parameter and it has been incorporated in both guidelines mentioned above. Recent findings indicate that HCM patients exhibiting non-extensive LGE, the involvement of subendocardium, rather than the extent of LGE, is linked to unfavorable outcomes [40]. For HCM patients without a defibrillator, CMR should be repeated every 3–5 years to monitor the progression of LGE and reassess strategies for preventing SCD [37].

### 2.4. Dilated Cardiomyopathy

Dilated cardiomyopathy (DCM) is the phenotypic description for a group of heart diseases associated with increased enddiastolic volume, reduced ejection fraction and usually increased filling pressures. The causes are very diverse and can be genetic, inflammatory, toxic, autoimmune, metabolic, or associated with neuromuscular diseases or congenital heart defects. The dilated form of ischemic cardiomyopathy should not be referred as DCM. The dilatation itself may reflect the final stage of a previously non-dilated cardiomyopathy, which makes identification using LGE at early stages particularly important. The new entity-term “non-dilated left ventricular cardiomyopathy” is discussed separately below.

LGE in DCM is often located in the mid-wall of the basoseptal segments of the heart but can have a variety of patterns (Figure 1E). The presence of LGE in DCM patients varies from 21% to 70%, and showed an averaged occurrence of 44% in a large meta-analysis [41]. The so called “ring-like” pattern especially is gaining in importance lately [42,43] (Figure 1F), because the distribution allows conclusions about the etiology and therefore leads to risk stratification.

LGE is able to identify fibrosis and the microstructure of fibrosis has been observed to influence electrical reentry [44]. Understanding these pathomechanism can enhance risk stratification and inform decisions regarding treatment. LGE has been related to adverse clinical outcomes in a large number of patients with DCM [41,45,46,47,48]. T1 mapping and ECV may have limited value in DCM, attributing this limitation to reduced accuracy caused by myocardial thinning [49].

### 2.5. Non-Dilated Left Ventricular Cardiomyopathy

The self-titled “major innovation” of the ESC guideline for cardiomyopathies [1] is the implementation of tissue characterization by LGE in CMR. For the new recognized phenotype, the term non-dilated left ventricular cardiomyopathy (NDLVC) is used, “defined as the presence of non-ischaemic LV scarring or fatty replacement regardless of the presence of global or regional wall motion abnormalities, or isolated global LV hypokinesia without scarring” [1].

CMR with LGE is the only way to presume a specific NDLVC, except for genetic testing, which is generally not carried out in previously asymptomatic or less symptomatic individuals. The prognostic significance varies with the underlying etiology. Some specific genotypes are associated with an increased risk of life-threatening arrythmias, e.g., LMNA mutation [50]. The LGE pattern cannot be used to draw absolutely certain conclusions about the underlying mutation, but it can help to guide genetic diagnostics and identify patients at high risk at an early stage. As mentioned above, the ring-like or nearly ring-like pattern gains special importance (Figure 1F): For example, mutations in the gen of filamin C, desmoplakin and phospholamban frequently show a subepicardial, ring-like pattern. Titin, laminin A/C and genotypes of Duchenne muscular dystrophy often show less or even no scar, with a more septal or inferolateral localized pattern and a more severe kinetic dysfunction [1,43]. It becomes clear that the total burden of LGE must be considered in relation to the underlying disease in order to assess the risk.

### 2.6. Arrhythmogenic Cardiomyopathy

Arrhythmogenic cardiomyopathy (ARVC) is a genetic disorder characterized by replacement of myocardium by fatty and fibrous tissue, frequent right ventricular enlargement, dyskinetic aneurysms and occurrence of ventricular arrhythmias. The International Task Force published the updated diagnostic criteria, called the Padua-criteria in 2020, which take the possible involvement of the left ventricle and the role of CMR more into account [51]. The ESC guidelines do not recommend the recently used term arrhythmogenic cardiomyopathy (ARC), as it lacks a morphological or functional definition [1]. It has to be mentioned that fatty replacement is not specific for ARVC, and diagnosis cannot be made only by CMR. Right ventricular ejection fraction measured by CMR is the only influencing factor in the ARVC calculator (ARVC Risk Calculator) until now.

Depending on the affected area, LGE can present with a patchy distribution pattern, especially in areas of dyskinesis and thinning (Figure 1G). Previously, the so-called “triangle of dysplasia” (RV outflow tract, RV cardiac apex, subtricuspid region of the free RV wall) was assumed to be the predilection site. Today we know from CMR studies that up to 76% of ARVC subjects have left ventricular involvement [52]. The LGE usually affect the inferior and lateral walls of the LV without abnormal wall motion. Although the presence and extent of LGE has been shown to be associated with poor prognosis [52,53,54], because LGE reflects the arrhythmogenic substrate of ventricular arrhythmia, is not yet fully established as a risk marker.

### 2.7. Cardiac Amyloidosis

In cardiac amyloidosis (CA) there is an expansion of the intercellular space due to deposition of amyloid fibrils, mainly Transthyretin (ATTR) or immunoglobulin-derived light chains (AL), which lead to pseudo-hypertrophy of the myocardium [55,56]. Hypertrophy often extends to the right ventricle, the atria and the interatrial septum, including thickening of the valves. This leads to a predominant diastolic dysfunction with typical symptoms of venous congestion.

Corresponding to hypertrophy, patients show a very characteristic pattern of LGE, which begins diffuse subendocardial in all affected parts of the heart (Figure 1H). In many patients a typical so-called zebra pattern of LGE distribution can be found with a subendocardial and an epicardial hyperintense line separated by a mid-myocardial hypointense zone. A transmural, strong enhancement occurs in advanced stages, what makes it difficult to null the myocardium in the T1-weighted inversion recovery sequences for LGE and simultaneously utilize this special phenomenon for diagnosis [57]. The extent of LGE correlates with the burden of disease, so LGE is a risk marker of disease progression [58]. Because LGE burden as a visual parameter is difficult to assess in small changes, CMR techniques such as pre-/post-contrast T1-mapping with subsequent ECV calculation, which is based on the extent of Gadolinium uptake, is more sensitive to monitor CA [59,60]. CMR has becoming increasingly important in diagnostics [55]. Risk stratification based on LGE/T1/ECV values is not yet a standardized clinical tool, but it is still used in individual cases to monitor follow-up and decide on therapy in expert centers. Several studies found a strong correlation between mortality and gadolinium uptake [61,62,63].

### 2.8. Fabry Disease

Fabry disease is an X-linked lysosomal storage disorder with a hypertrophic phenotype, which arises from the accumulation of glycosphingolipids and the hypertrophy of myocytes [64]. The early stage of the disease typically shows reduced T1 mapping values, which distinguishes Fabry disease from the most other hypertrophic phenotypes. In the course of the disease, increasing fibrosis can lead to a pseudo-normalization of the values.

LGE is often located in the inferolateral segments of the LV basis with a mid-mural to subepicardial deposition [39] (Figure 1I). The LGE can even be there in mutation carriers without the presence of hypertrophy. It is a risk marker for poor response to enzyme replacement therapy with alpha-galactosidase and associated with adverse outcome [64].

### 2.9. Endomyocardial Fibrosis

Endomyocardial fibrosis (EMF) shows an apical hypertrophy due to thickening of the endocardium by deposition of fibrous tissue. EMF is not sufficiently recognized in western countries, because it is predominant in tropical regions [65]. The causes are divers, e.g., expose to toxic agents or inflammation, such as Löffler endocarditis, the cardiac involvement in hypereosinophilia syndrome. This leads to reduced end-diastolic volume with symptoms of diastolic dysfunction.

CMR with LGE is considered the gold standard for evaluating EMF, particularly for the localization, characterization, and quantification of fibrous tissue. LGE lies in the thickened subendocardial layer, apically emphasized (Figure 1J). An apical thrombus is often present. LGE strongly correlates with histopathological findings, and the extent of LGE is associated with an increased risk of mortality [66].

### 2.10. Cardiac Sarcoidosis

Sarcoidosis is a special case of inflammation, which has to discussed separately. It is an inflammatory, granuloma-forming disease of unknown origin, which is mainly characterized by involvement of the lungs and mediastinal lymph nodes. Cardiac involvement is found in 10–25% of all sarcoidosis patients and can also occur in isolation in individual cases. Involvement of the left ventricular myocardium and the conduction system is predominant, so that affected patients often present clinically with cardiac arrhythmias and/or heart failure symptoms [67,68]. In addition to clinical, laboratory and nuclear examinations, CMR has a special role.

LGE often shows a typically multifocal, patchy pattern, which can lead to the visual diagnosis of cardiac sarcoidosis (Figure 1K). The LGE cannot be assigned to any coronary area, occurs subendocardially, subepicardially, and also transmurally, and often shows an impressive intensity, which contrasts with an often preserved function. The basal septum or the anteroseptal wall with affection to the RV (sometimes called “hook sign”), the basal inferolateral wall and the inferior RV insertion (“triangle sign”) are predicating sites [68]. Cardiac involvement has significant prognostic importance. According to the guidelines, primary prophylactic implantation of an ICD should be considered if there is a pronounced scar on CMR, regardless of the LVEF [69].

### 2.11. Neuromuscular Diseases with Cardiac Involvement

Neuromuscular diseases present with symptoms affecting the skeletal muscle and show a varying prevalence of cardiac involvement, which significantly affects mortality. Phenotypically, a DCM or NDLVD may be present.

LGE with subepicardial involvement of the LV lateral free wall was found to be the most frequent pattern in muscular dystrophy [70] (Figure 1L). Mitochondriopathies show a completely different pattern, for example in MELAS (Mitochondrial encephalopathy, lactic acidosis, and stroke-like episodes) LGE is focally accentuated and diffusely distributed [71]. The presence of LGE indicates individuals who are at risk of developing progressive left ventricular dysfunction and was also linked to a heightened risk of mortality [72].

### 2.12. LGE for Further Cardiac Diseases

The presence of LGE has been recognized in several other cardiological conditions. It has been used in some cases for diagnostic and prognostic purposes, but so far it has been of secondary importance or not fully understood. For example, congenital heart disease or acquired valve disorders may develop fibrosis due to abnormal filling pressures or surgical scars. LGE was a powerful predictor of all-cause mortality in patients with aortic stenosis [73], but this does not currently play a major role in the evaluation of patients. Non-compaction cardiomyopathy usually shows no or few LGE but small studies suggest the value of LGE risk stratification in this patients [74], although a lower sensitivity must also be assumed here with thinner myocardium. Takotsubo syndrome usually shows no or rapidly transient LGE. Takotsubo or myocarditis illustrates that in reversible damage of the myocardium, LGE or a part of the full extent of LGE is transient. The prognostic significance of the transient detection of LGE has not yet been sufficiently researched.

## 3. Discussion

CMR is characterized by its ability to provide precise anatomy and functional evaluation. In addition, it enables early detection and differentiation of various cardiomyopathies through high spatial resolution and tissue characterization capabilities, implemented by differently weighted sequence types and the use of the MRI contrast agent gadolinium. LGE imaging has proven to be a valuable marker for risk stratification in various heart diseases. LGE should not be considered in isolation, as other CMR techniques are of great importance, such as cine-imaging as the gold standard for right and left ventricular ejection fraction assessment, native and post-contrast T1 mapping for quantification of even subclinical tissue alteration, T2-weighted images to assess oedema, or stress perfusion imaging as a CMR-guided selection strategy for revascularization.

The patient evaluation should include the medical history, family history, physical examination, electrocardiographic patterns, and transthoracic echocardiography (TTE) as the gold standard of cardiological imaging prior to CMR. It must be emphasized that a comprehensive medical history including extracardiac symptoms is essential for the correct interpretation of CMR images. TTE is widely available, easy to use and is primarily used for anatomical and functional assessment. In some cases, e.g., evaluation of valve stenosis and assessment of diastolic dysfunction, it is superior to CMR. TTE plays an important role in the initial assessment and in raising diagnostic suspicions, but its utility becomes limited when establishing differential diagnosis, based on tissue characterization.

There are some difficulties in establishing LGE as a risk marker, using HCM as an example: first, observational studies have identified several risk factors for sudden cardiac death in HCM but individually, these factors exhibit a low positive predictive value [2]. Efforts to establish the prognostic significance of LGE for sudden cardiac death in HCM patients have been hampered by similar problems, namely the relatively low event rates [75]. The prevalence of LGE is much higher, than the rate of adverse events. Therefore, relatively small study populations do not have sufficient statistical power. At least a meta-analysis of nearly 3000 patients showed that LGE is associated with a 2,3-fold increased risk of SCD [76], so LGE has been suggested to improve risk stratification [37]. Now it is recommended in the ESC guidelines for patients who are in the low to intermediate risk category (by first using the HCM risk calculator) to improve decision-making about prophylactic ICD-implantation [1,2] (IIa/B [2], IIb/B [1]). Second, several studies observed that maximum wall thickness was significantly higher in patients with LGE compared to those without LGE [77,78]. Consequently, all patients with a wall thickness exceeding 30 mm, an established risk factor for sudden death, were exclusively present in the LGE groups in those studies. So, whether LGE is an epiphenomenon of advanced cardiac remodeling that correlates with existing prognostic markers (e.g., myocardial mass) and is thereby confounded, or whether it has independent prognostic significance, is not yet fully understood. Third, there can be another confounder, namely the technical method of interpretation because LGE quantification dependents on CMR acquisition, type, and amount of contrast. In clinical routine LGE is assessed visually, which clearly depends on subjective judgement and personal experience but is not inferior to quantitative measurement when assessed by an experienced physician. To quantify LGE, the 2-standard deviation technique is the only one validated against histological examination [79]. A generally accepted, standardized method for the quantification of fibrosis in CMR has not yet been established.

Prior problems of CMR have been solved in current times: nephrogenic systemic fibrosis as a rare complication of linear unstable gadolinium chelates of the first generation in patients with severe renal impairment. It is practically not reported with the use of newer, macrocyclic gadolinium contrasts. Modern Gadolinium-based contrast agents can be safely utilized for patients with an estimated glomerular filtration rate > 30 mL/min/1.73m^2^. Depending on the urgency and under strict precautions, the use of gadolinium based contrast agent is possible even in patients requiring dialysis [80,81,82]. In addition, there have been limitations in imaging patients with cardiac implantable electrical devices (CIEDs) due to safety concerns and image artefacts. Today, there are solutions to reduce the artefacts, such as wideband sequences for LGE imaging, so CMR is possible even in patients with a large subcutaneous implantable cardioverter defibrillator [83]. In addition, MR-conditional devices have been available for around 10 years and data on formally non-conditional CIEDs are comprehensive and still growing [84].

The upgrading of the CMR in the guidelines is certainly to be encouraged. However, this also means that non-implementation of a CMR will increasingly have to be regarded as non-compliance with the guideline. In reality, access to CMR in many regions is often limited by cost and reimbursement, as well as by the qualifications of the assessing physicians and the lack of training centers. For this reason, widespread availability and broad application would be desirable to gather individual and scientific experience.

However, again in a large meta-analysis with almost 8000 patients and a heterogenous spectrum of cardiovascular diseases LGE was strongly correlated with various adverse outcomes, including all-cause mortality (HR 2.96, 95% CI: 2.37, 3.70, *p* < 0.001), cardiovascular mortality (HR 3.27, 95% CI: 2.05, 5.22, *p* < 0.001) and ventricular arrhythmia/sudden cardiac death (HR 3.76, 95% CI: 3.14, 4.52, *p* < 0.001) [85]. In addition, LGE was associated with mortality in both, left ventricular ejection fraction under and over 35%, and this in individuals with nonischemic and ischemic cardiomyopathy. In conclusion, CMR has proven as robust marker of adverse outcome in cardiovascular disease of different origin.

## 4. Limitations

It should be noted that LGE accumulation is not typical for all cardiac diseases. Typically, channelopathies show no conspicuous features in the CMR, including no LGE. CMR has proven as an outstanding screening and therapy monitoring tool for cardiac iron overload, showing characteristically lowered T1 and T2 * values [86], but LGE imaging is not relevant here.

There is limited diagnostic specificity concerning LGE alone: similar distribution patterns can indicate very different diseases (e.g., basal, subepicardial, inferolateral LGE in muscular dystrophy Duchenne or myocarditis). A great deal of experience and the overall medical context are therefore important for the interpretation of the images.

The sensitivity of the method is also limited because it depends on the severity of the disease, i.e., the degree of cell destruction or replacement. So, the absence of visible LGE does not exclude the diagnosis. Multi-parametric CMR, especially the more sensitive T1 mapping, is recommended to overcome this problem. However, this method is not available in all centers due to a lack of software solutions and has not yet been sufficiently validated for all diseases.

## 5. Conclusions

LGE in CMR has proven as a valuable tool for diagnosis and beyond that as a reliable risk marker of adverse outcome in cardiovascular disease of different origin. Compared to the pre-CMR era, risk acquisition and stratification needs to be rethought nowadays. CMR with LGE should be offered in every initial assessment of cardiomyopathy, as recommended in the guidelines. As a result, early diagnosis, correct risk assessment, and targeted therapy will have an impact on survival rates.

## 6. Future Directions

Instead of considering CMR as a reserve diagnostic for special cases, it will be necessary to integrate it more into everyday life. Widespread availability and application would be desirable to gain individual and scientific experience.

## Data Availability

Publicly available datasets were analyzed in this study. This data can be found here: https://pubmed.ncbi.nlm.nih.gov/ (accessed on 1 December 2023).
